# Accelerated Care of Patients with Hip Fractures is Associated with Lower Risk of Delirium and Infection, and a Shorter Length of Hospital Stay: Systematic Review and Meta-analysis of Level One Evidence

**DOI:** 10.1007/s43465-023-01026-x

**Published:** 2023-11-16

**Authors:** P. Shah, E. Wilson, B. Chen, N. D. Clement

**Affiliations:** 1 University of Edinburgh Medical School, 47 Little France Cres, Little France, Edinburgh, EH16 4TJ UK; 2 Department of Orthopaedics, Second Affiliated Hospital of Soochow University, Suzhou, China; 3 Edinburgh Orthopaedics, Royal Infirmary of Edinburgh, Little France, Edinburgh, UK

**Keywords:** Hip fracture, Time to theatre, Accelerated surgery, Perioperative outcomes, Length of hospital stay, Mortality, Delirium, Infection

## Abstract

**Objectives:**

The aim of this systematic review was to assess the impact of time to surgery on patient mortality, peri-operative complication rates and length of stay following a hip fracture using level one data.

**Data Sources:**

Multiple databases (PubMed, Embase, Medline (Ovid), and Cochrane Library) were searched using terms for “hip fracture” and the intervention “early surgery”. Results were filtered to only included randomised controlled trials in the English language published from the year 2000.

**Study Selection:**

All results were imported into Covidence and screened by two separate reviewers with conflicts resolved by a third reviewer. Studies were included if they reported data on the relationship between time to theatre and at least one of the outcome measures (mortality, peri-operative complications, and length of stay in hospital). Three papers were finalised to include in this review.

**Data Extraction:**

Once selected, each paper had a bias assessment completed by two separate reviewers using the Cochrane RoB2 tool. Any conflicts were resolved by a third reviewer.

**Data Synthesis:**

Data from each paper were inputted into RevMan5 for analysis. Approximated sample mean and standard deviation were collected from each paper and included for analysis. RevMan5 was then used to generate forest plots and report data on relative risk and mean difference.

**Conclusions:**

This review has shown that accelerated care of patients with hip fractures was associated with lower risks of delirium and infection, and a shorter length of hospital stay. However, the effect of time to surgery on patient mortality is not clear, as the standard care group had a lower mortality than expected for the population at risk and had surgery on average within 24-h of presentation.

## Introduction

The worldwide lifetime risk of a hip fracture for women is 17.5% and 6% for men with an average mean age of 77 and 72 at time of injury, respectively [1, 2]. Decreasing bone mass and increasing incidence of falls result in an exponential increase in hip fractures with aging [3]. By the age of 80, approximately 17% of males have suffered from a hip fracture [4] and that number increases in females to approximately 33%.

Early surgical repair of a hip fracture is associated with lower mortality and complication rates [1, 5]. Some studies have suggested that having patients undergo surgery within a certain period following the fracture has the potential for better outcomes [6–14]. However, studies demonstrating these rates do not account for the possibility that the delay to surgery is more likely in patients who are inherently comorbid and may bias such a comparison simply based on time to theatre. As patients that have their surgery delayed for medical optimisation may have a higher likelihood of complications and mortality following surgery [15]. The ‘Management of Hip Fracture in Adults’ guideline produced by the National Clinical Guideline Centre in 2020 stated that the evidence for early surgery showing statistically and clinically significant reduction in mortality and complications is ‘low quality’ or ‘very low quality’ [16]. A recent systematic review looking at the impact of time to surgery on hip fracture patients by Klestil et al. in 2018 [17] was unable to conduct any analyses on various subgroups regarding differing time to theatre, including age, sex, physical status, and anticoagulation. They stated “in healthy, independent patients, delayed surgery was not as problematic as in patients with comorbidities”. All systematic reviews and meta-analyses to date have included level three retrospective comparative cohort studies and none have included randomised controlled trials (RCTs) only (26, 27). Among these observational studies, cohorts have been arbitrarily divided up into either ≤ 24 h and > 24 h, or ≤ 48 h and > 48 h to observe outcomes. Such a study design is flawed as patients who have delayed surgery are likely those needing medical optimisation prior to surgery, creating an allocation bias.

The aim of this review was to assess the impact of time to surgery on patient mortality following a hip fracture using data from RCTs only. The review also assessed the impact of time to surgery on peri-operative complication rates and length of stay in hospital.

## Methodology

To answer our research questions, we conducted a systematic review into all randomised controlled trials available. The protocol for this review has been registered with International prospective register of systematic reviews (PROSPERO), registration ID: CRD42022284088. The important steps in the methodology are outlined below.

### Search Strategy

The literature search was done using the following databases: PubMed, Embase, MEDLINE (Ovid), and Cochrane Library. The search strategy was largely based on the one done by Klestil et al. (2018) [17], outlined in their study protocol.

Search terms included terms for the condition (hip fracture) and the intervention in question (early surgery). We considered all papers published in the English language from the year 2000. To focus the search, we also filtered search criteria for RCTs.

**Fig. 1:**
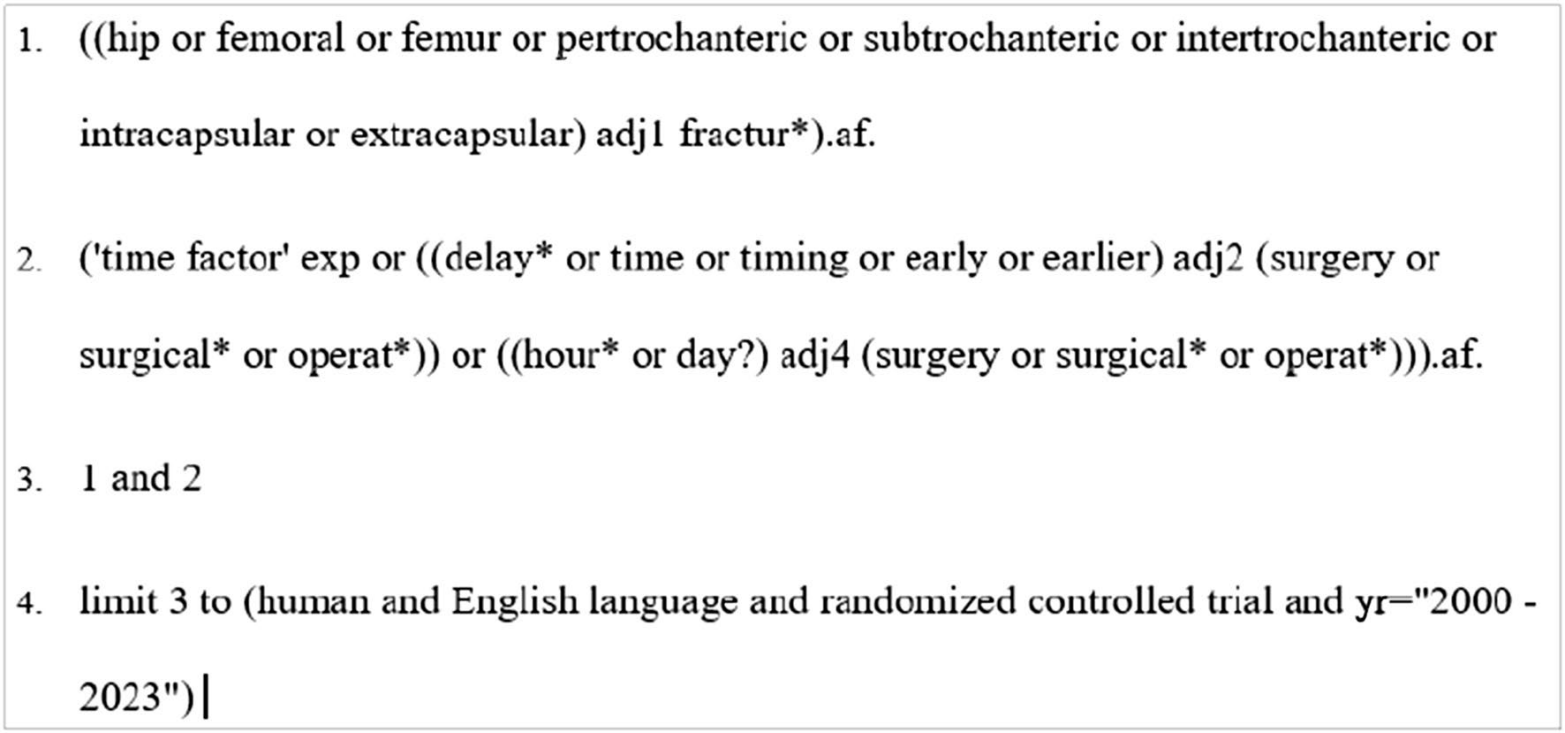
Embase search strategy

The search strategy for Embase is shown in Fig. 1.

The literature search was done on the 7th of November 2021 and papers identified were transferred into Endnote. In total, there were 633 results from PubMed, 235 results from Embase, 178 results from MEDLINE, and 918 results from Cochrane Library (70 systematic reviews and 848 clinical trials). The final collection of papers was then transferred into Covidence which removed all duplicates to give a final number of 1324 papers for screening.

### Study Selection

Papers were screened on Covidence by two independent reviewers and all conflicts were resolved by a third reviewer. Titles and abstracts were screened initially, followed by full text screening.

Randomised controlled trials in the English language which looked at the relationship between time to theatre and at least one of the outcome measures (mortality, peri-operative complications, and length of stay in hospital) were included. Non-English papers and studies looking at paediatric populations were excluded, as were studies of the wrong study design (e.g., cohort studies, audits, case studies, etc.).

### Quality Assessment

Bias assessments were done using the Cochrane RoB2 tool [18], provided on Covidence, by two independent reviewers. Any conflicts in the decisions were resolved by a third independent reviewer.

### Data Extraction and Analysis

Once papers were finalised for review, data were inputted into RevMan5 for analysis of effect estimate as appropriate. Determination of use of random effects or fixed effects model was determined by heterogeneity of data, calculated by the *I*^2^ statistic [19]. For uniformity in the values and data reported throughout all the papers, approximation methods for sample mean and standard deviation were used [20]. Larsson et al. [21] reported mean values, however, to maintain consistency in the meta-analysis, the mean and standard deviation were approximated using the equations outlined in Wan et al. [20].

## Results

**Fig. 2:**
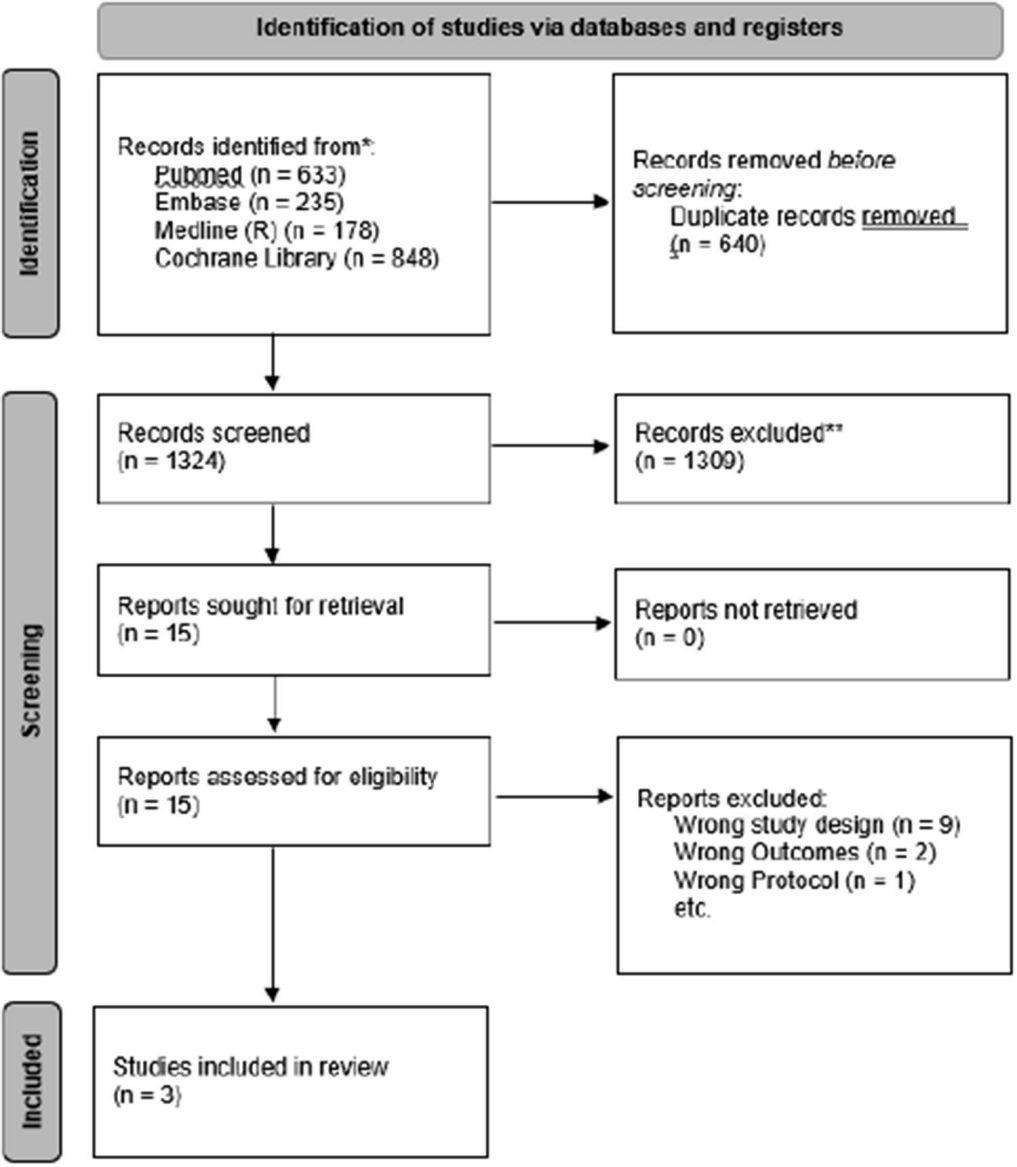
PRISMA flow chart

The literature search identified 1964 studies in total. After importing them into Covidence, 640 studies were automatically removed as duplicates leaving 1324 studies for the 2 reviewers to screen. After the initial abstract and title screening, 1309 studies were removed leaving 15 studies for full text screening. Out of these, 12 studies were excluded for either the wrong study design, the wrong outcomes, or for being a study protocol. The remaining three studies were included in the systematic review. The full screening process can be seen in the PRISMA chart (Fig. 2).

The included studies provided data on 3430 patients. Table 1 shows the demographics and other characteristics of each study.

**Table 1:** Study characteristics

Author, year of publication	Sample size	Age, mean (SD or Range)	Female %	Comparators	Primary outcomes
Larsson, 2016 [21]	400 (195 in PFTC Group; 205 in A&E Group)	PFTC: 83 (53–103) A&E: 82 (50–99)	PFTC: 68% A&E: 66%	Prehospital fast track care vs A&E	Time to radiographic imaging; Time to surgery; Length of stay at hospital; Mortality
HIP ATTACK Investigators, 2014 [22]	60 (30 in accelerated care group; 30 in standard care group)	Accelerated care: 80 (10) Standard Care: 80 (9)	Accelerated care: 57% Standard Care: 70%	Accelerated care vs standard care	Perioperative complications within 30 days of randomisation
HIP ATTACK Investigators, 2020 [23]	2970 (1487 in accelerated surgery group; 1483 in standard care group)	Accelerated surgery: 79 (12) Standard care: 79 (11)	Accelerated care: 69% Standard care: 69%	Accelerated care	Mortality at 90 days; Composite of major complications at 90 days

### Time to Theatre

**Fig. 3:**
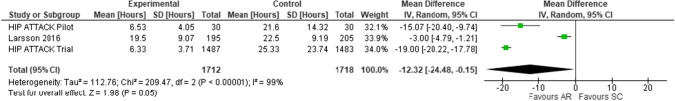
Time to theatre

Each of the three studies randomized their patients into either an accelerated or standard route of care for the patient’s hip fracture. There were no clear allocations made for time to surgery, although the differences between the two groups can be seen in Fig. 3.

Meta-analysis showed that patients allocated to the accelerated route had their surgery significantly quicker than patients allocated to the standard care route (Mean difference − 12.32 h, 95% CI − 24.48, − 0.15, *p* < = 0.05). Both HIP ATTACK trials [22, 23] had a much quicker time to theatre compared to the trial done by Larsson et al. [21] (− 3 h), and the time differences between the different comparator groups was more pronounced in the HIP ATTACK [22, 23] trials (− 15.07 and – 19 h, respectively).

### Mortality

**Fig. 4:**
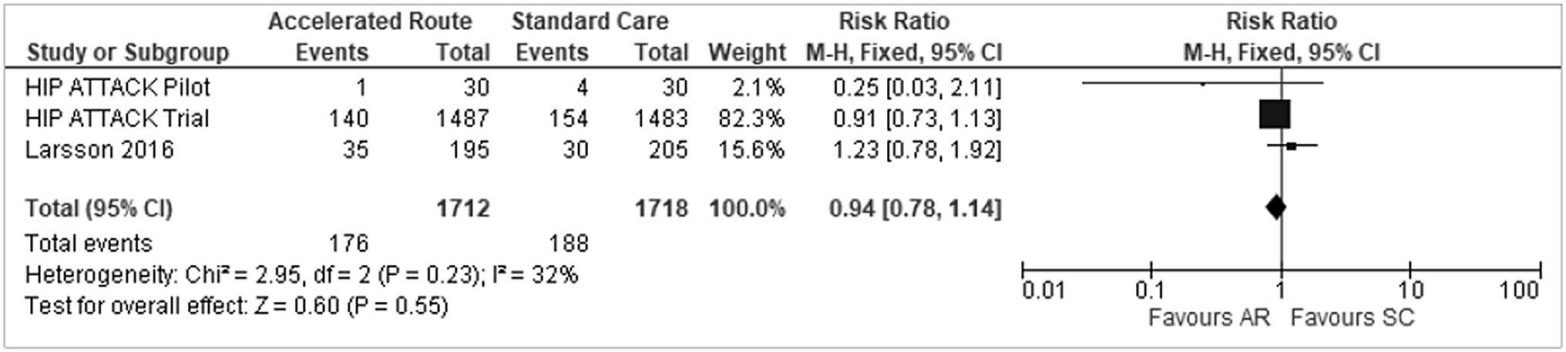
Mortality

All three studies assessed mortality as their primary outcome measure. Meta-analysis demonstrated no statistically significant difference in mortality rates between either group (RR 0.94, 95% CI 0.78, 1.14, *p* = 0.55) (Fig. 4). However, there was a lower mortality rate (0.66% absolute risk reduction) observed in the accelerated group compared the standard care group (10.28% versus 10.94%). Each trial assessed mortality at different time intervals: 30 days [22], 90 days [23], and 4 months [21], but for the purposes of this meta-analysis, all the data were pooled for assessment.

### Peri-operative Complications

**Fig. 5:**
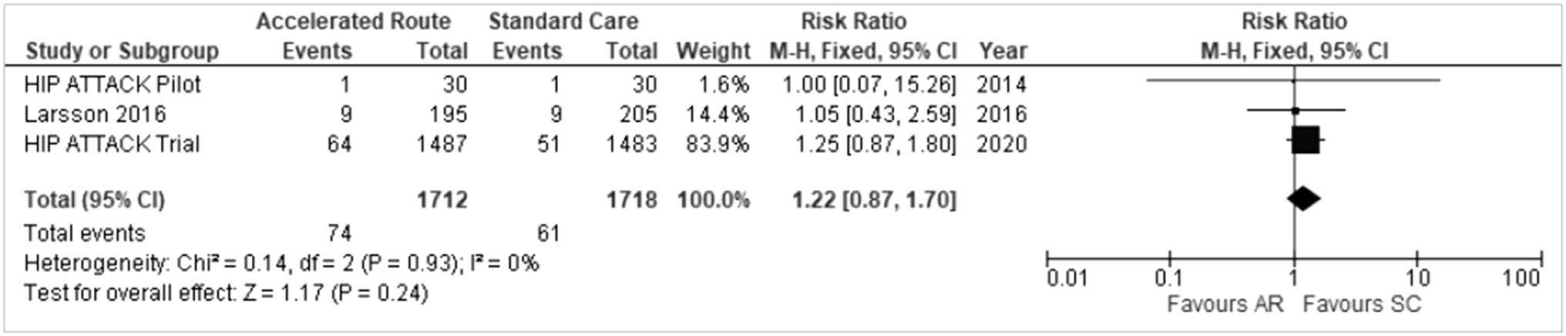
Pneumonia

Each study recorded peri-operative complications as an outcome measure, however, not all studies included the same complications. The only complication that was common between the three studies was cases of pneumonia. Meta-analysis of risk of pneumonia (Fig. 5) found no statistically significant difference between either groups (RR 1.22, 95% CI 0.87, 1.70).

**Fig. 6:**
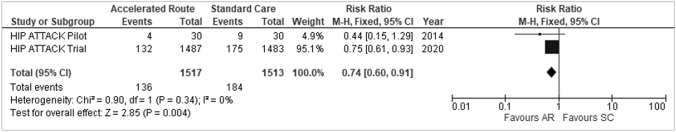
Delirium

**Fig. 7:**
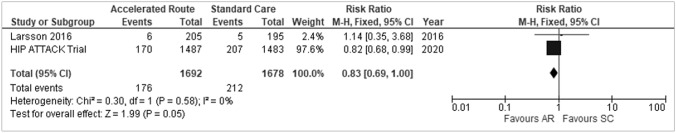
Infection (not defined in papers)

**Fig. 8:**
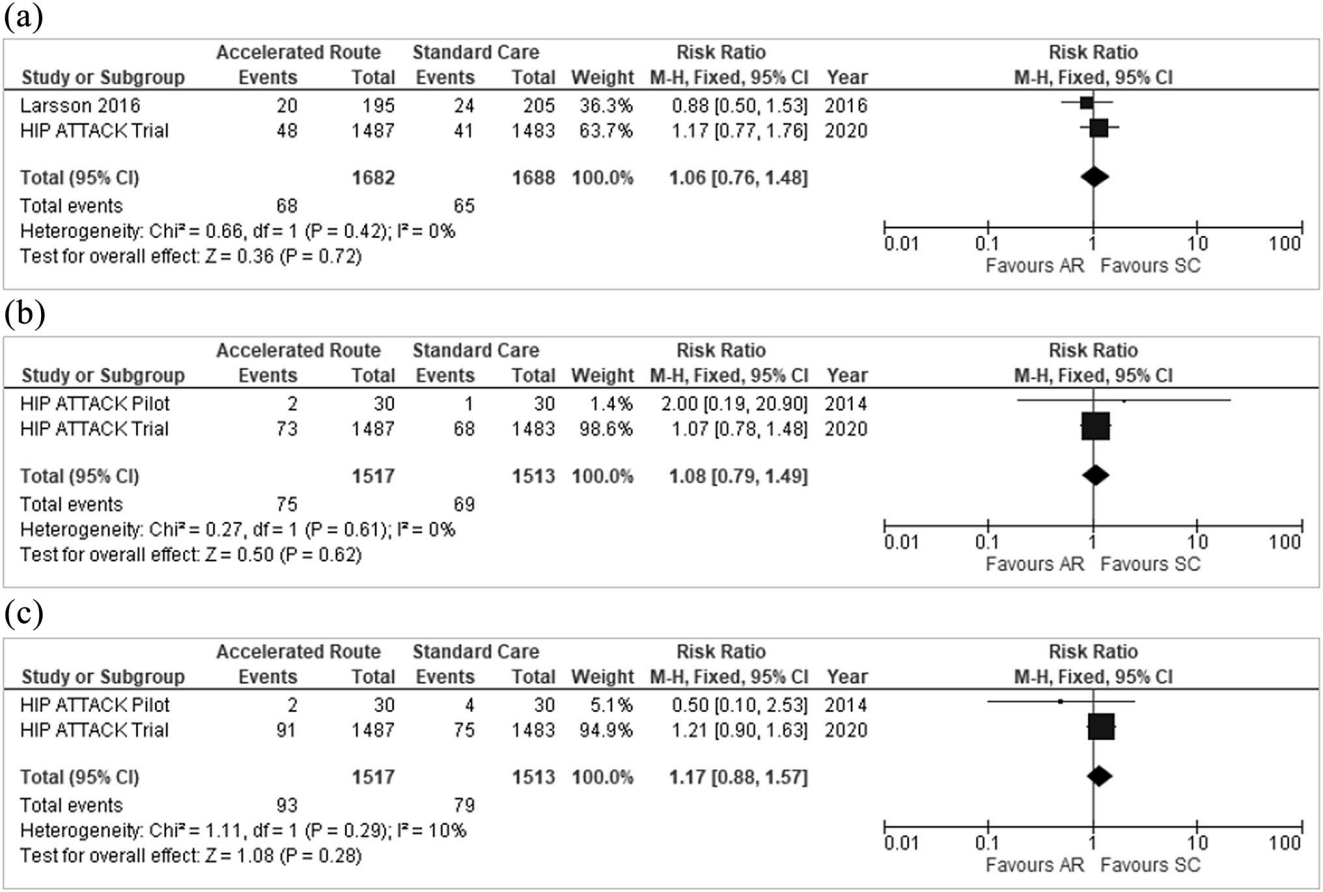
**a** Pressure ulcers, **b** Sepsis, **c** Major bleeding

There was a significantly lower risk of delirium (RR 0.74, 95% CI 0.60–0.91, *p* = 0.004) (Fig. 6) and infection (RR 0.83, 95% CI 0.69–1.00, *p* = 0.05) (Fig. 7) in the accelerated group. There were no differences observed in the other peri-operative complications assessed: pressure ulcers (RR 1.06, 95% CI 0.76, 1.48), sepsis (RR 1.08, 95% CI 0.79, 1.49), or major bleeding (RR 1.17, 95% CI 0.88, 1.57) (Fig. 8a–c).

### Length of Hospital Stay

**Fig. 9:**
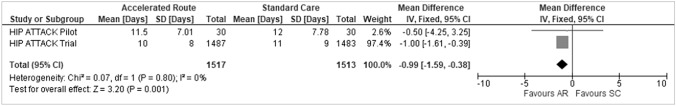
Length of hospital stay (days)

The HIP ATTACK trials [22, 23] looked at the differences in length of stay in hospital as an outcome measure and meta-analysis showed a statistically significant difference favoring patients in the accelerated group (Mean Difference − 0.99, 95% CI − 1.59, − 0.38, *p* = 0.001) (Fig. 9), meaning that patients in the accelerated care pathway stayed in hospital for a shorter duration than patients in the standard care pathway.

### Bias Assessment

The results of the Cochrane RoB2 bias assessment of the included reviews is seen in Table 2.

**Table 2:** Bias assessment of studies

	Sequence generation	Allocation concealment	Blinding of participants and personnel	Blinding of outcome assessors	Incomplete outcome data for all outcomes	Selective outcome reporting	Other sources of bias
HIP ATTACK Pilot (2014)	Low	Low	Unclear	Unclear	Low	Low	Low
Larsson (2016)	Low	Low	High	High	Low	Low	High
HIP ATTACK (2020)	Low	Low	High	Low	Low	Low	Low

## Discussion

This review has shown that accelerated care of patients with hip fractures results in lower risk of delirium and infection, and a shorter length of hospital stay. Although the accelerated care pathway was associated with a 0.66% lower mortality risk, this was not statistically significant. This review did not aim to assess accelerated care pathways for management of hip fracture patients and was aiming to be inclusive of all RCT’s comparing time to theatre, however, the only currently available evidence was for accelerated pathways versus standard care. Of note, two of the three included studies had the same design, with one being the pilot trial. The HIP Attack pilot trial showed the feasibility of the trial design, allowing the HIP Attack trial to be conducted. While the trial designs remained similar, there was no data duplication as any patients previously enrolled, including the pilot study, were excluded from the HIP Attack trial.

It would seem intuitive that shorter time to surgery would have a beneficial effect on a patient’s outcome and result in a lower mortality rate, however, this was not the case according to the results from current review. There are several factors that may explain this. The HIP Attack study was powered to show a reduction in 90-day mortality, which was assumed to be 13% for standard care pathway, with a hazard ratio of 0.7 which equates to approximately a 30% reduction i.e., an absolute risk of 9%. The observed mortality rate in the accelerated care group was 9%, the same as predicted, but the reason they did not show a significant difference was likely due to a lower than expected mortality in the standard care group of only 10%. The reason for the lower than observed mortality rate in the standard care group may relate to the Hawthorne effect, which is recognised to have a positive influence on outcome [24], and the precise inclusion criteria with 27,701 being screened but only 2870 were enrolled. To demonstrate the 0.66% difference in mortality between accelerated and standard care 68,354 patients would be needed to be randomised to achieve 80% power with an alpha of 0.05, which may not achievable. Another reason why no difference was observed in mortality may be the relatively short time to theatre observed in the standard care group of only 24 h from time of hip fracture diagnosis. Therefore, there may be a ceiling to the benefit of an accelerated pathway, as it is well recognised that longer than 24 h to theatre is associated with a significantly greater mortality risk. [25].

One of the novel aspects of this review is the significantly lower risk of delirium, reduced by approximately 25%, when accelerated care pathway was employed compared to the standard care. This finding is consistent with a recent review which showed delay of care of more than 48 h being associated with twice the risk of developing delirium [26]. The prevalence of delirium in patients following hip fracture has been reported to be as high as 50% postoperatively which has repercussions on their outcome [27]. Developing postoperative delirium is associated with poorer functional recovery, greater risk of readmission and reoperation, and higher postoperative complications and mortality [28]. Therefore, any intervention to reduce the risk of postoperative delirium, such as accelerated care, may improve the outcome for the patient.

The other complication that was significantly reduced with an accelerated care pathway was infection risk, however, it was not always clear from the included studies as to the definition of infection. The largest study being HIP ATTACK [23] more specifically looked at the risk of urinary tract infection (UTI) and demonstrated a significantly lower risk associated with accelerated care group (*n* = 120 [8%] patients) compared to the standard-care group (*n* = 150 [10%] patients), with a hazard ratio of 0.78 and absolute risk reduction of 2%. UTI is associated with sepsis, postoperative length of stay beyond 2 days, and hospital readmission. [29]. A recent review of 46,263 patients with a hip fracture identified that a delay of 2 days from admission to surgery was associated with an increased risk of developing a UTI (odds ratio = 1.37, 95% CI = 1.05–1.79). The current study suggests that even a shorter time to surgery was associated with a reduced risk of UTI and, therefore, potentially avoiding the morbidity associated with a UTI following a hip fracture. However, in contrast to the previous literature, despite the lower risk of UTI observed in the accelerated care group, no increased risk of sepsis was found either in the current study or the HIP ATTACK study [23].

The current study demonstrated a 1-day reduction in length of hospital stay associated with accelerated care pathway, relative to standard care, which may have economic benefits for healthcare systems employing this pathway. The average cost in the UK of an acute trauma bed for a patient with a hip fracture is £630 [30] and, therefore, an accelerated care pathway would be associated with an equivalent saving. However, the cost of implementing the accelerated pathway would need to be less than the cost saving of £630 from the shorter length of stay to be cost effective. In addition, the cost of managing delirium and infection complications, being greater in the standard group, would also need to be acknowledged.

An RCT published by Swanson et al. [31] assessing the effect of an “early intervention program” on the length of hospital stay demonstrated an 11.5-day reduction relative to the standard care group. This study was not included in the current review as it was published before 2000, which was one of the exclusion criteria. This study supports the findings of this review, with a shorter length of stay being associated with earlier time to surgery, but the effect size of 11.5-days does seem to be greater than might be expected. Swanson et al. compered 38 patients in the early intervention group and 32 in the standard group, with a median time to surgery of 1-day and 2-days, respectively, and therefore, a 1-day delay to surgery after the first 24-h following admission does seem to have a greater effect on the patient’s length of stay. The reasons for the increase in length of stay observed by Swanson et al. [31] are not clear and may be related to postoperative complications such as delirium and infective complications demonstrated in the current review, that may have subsequently delayed their discharge. Swanson et al. [31] did, however, assess mortality rate and found a slightly lower rate in the early group (5.2%, *n* = 2/38, versus 6.1%, *n* = 2/33), but this was an “in-hospital” assessment, and in the knowledge that the early group was discharged 11.5-days earlier this may not be reliable assessment.

The original aim of this review was to look at the effect time to surgical intervention on early mortality, complications and length of hospital stay, however, the only available RCTs compared accelerated care with standard care. The standard care group had a mean of 24 h to theatre, but the literature would suggest that the mortality risk is not increased until after the 24 h from admission [25]. More recently, data from the Norwegian Hip fracture Register suggested that mortality was not influenced when total delay to surgery was less than 48 h, whereas delay beyond 48 h was associated with an increased risk [32]. This literature may be biased in their selection and assessment of patients and, therefore, the associated increased mortality risk may be confounded by the reason(s) for their delay to surgery such as medical complications. The aim of the current study was to try and assess the independent effect of time to theatre on outcomes, but due to the limited number of RCT’s and the fact that time to theatre in the standard care group was a mean of 24 h the true effect of surgical delay beyond this time is not clear. The current review has shown the benefit of accelerated surgery and it is likely, even in the knowledge of the confounded retrospective study design, that earlier time to surgery has significant benefits for patients with hip fractures. The optimal time to theatre is debateable but it would seem the earlier the better and where resources are available hip fractures should be surgery treated within 24 h of admission when there are no contraindications to surgery.

Due to the lack of available data, this study came with a set of limitations impacting any firm conclusions drawn from the results. In both HIP ATTACK trials [22, 23], patients as well as healthcare workers (surgeons, nurses, anaesthetists, etc.), were not blinded to allocation of patients. However, there was protocol to blind outcome adjudicators. In the Larsson trial, ambulance nurses were responsible for allocation of patients. There was scepticism by these nurses regarding the standard care pathway and the authors believe that this may have impacted allocation of patients. The other limitation of the current meta-analysis is the variability in sample sizes between the RCTs. With one study accounting for 86.6% of the overall sample size, there is likely to have been a heavy influence on results reported, limiting a proper meta-analysis. One of the difficulties in doing the meta-analysis in this review was the variability in follow-up between the studies for assessment of mortality, which ranged from 30 to 90 days, to 4 months. The differing endpoints make it difficult to do a comparison, however this review decided to pool the values to offer some sort of analysis. Finally, since time to theatre was reported as an outcome measure, instead of as a variable, there was no standardised way to compare the impact of time to theatre on mortality, post-operative outcomes, and length of hospital stay. Furthermore, even between accelerated route of care and standard route of care, there was variation in the time to theatre between studies making it difficult to standardise or compare outcomes. The significant heterogeneity in the data resulted in a random effects model being used for analysis. Despite the wider confidence interval produced from the use of this model, the results of the analysis were still significant (*p* = 0.05), supporting shorter time to theatre in the accelerated group. However, the small number of included studies creates limitations which need to be acknowledged. The use of a random effects model in this case makes it harder to be precise with inter-study variance, which can accentuate the biases within the studies [33]. Furthermore, although the use of random effects model makes it possible to extrapolate the results to a wider population, the small number of included studies makes this more difficult as there may not be sufficient information to correctly apply the results more broadly, affecting the width of distribution [19].

In conclusion, accelerated care of patients with hip fractures was associated with lower risks of delirium and infection, and a shorter length of hospital stay. However, the effect of accelerated care on patient mortality is not clear due to the potential selection bias of the available studies, with the standard care group having a lower mortality than expected for the population at risk.
