# Correction: Accelerated Care of Patients with Hip Fractures is Associated with Lower Risk of Delirium and Infection, and a Shorter Length of Hospital Stay: Systematic Review and Meta-analysis of Level One Evidence

**DOI:** 10.1007/s43465-024-01120-8

**Published:** 2024-02-22

**Authors:** P. Shah, E. Wilson, B. Chen, N. D. Clement

**Affiliations:** 1 University of Edinburgh Medical School, 47 Little France Cres, Little France, Edinburgh, EH16 4TJ UK; 2 Department of Orthopaedics, Second Affiliated Hospital of Soochow University, Suzhou, China; 3 Edinburgh Orthopaedics, Royal Infirmary of Edinburgh, Little France, Edinburgh, UK

**Correction: Indian Journal of Orthopaedics (2023) 58:1–10** 10.1007/s43465-023-01026-x

In this article the author’s name Panth Shah was incorrectly written as Shah Panth.

The original article has been corrected.

